# Sodium valproate-induced congenital cardiac abnormalities in mice are associated with the inhibition of histone deacetylase

**DOI:** 10.1186/1423-0127-17-16

**Published:** 2010-03-10

**Authors:** Gang Wu, Changlong Nan, Johnathon C Rollo, Xupei Huang, Jie Tian

**Affiliations:** 1 Department of Cardiology, the Children's Hospital of Chongqing Medical University, Chongqing, China; 2 College of Biomedical Science, Center for Molecular Biology and Biotechnology, Florida Atlantic University, Boca Raton, FL 33431, USA

## Abstract

**Background:**

Valproic acid, a widely used anticonvulsant drug, is a potent teratogen resulting in various congenital abnormalities. However, the mechanisms underlying valproic acid induced teratogenesis are nor clear. Recent studies indicate that histone deacetylase is a direct target of valproic acid.

**Methods:**

In the present study, we have used histological analysis and RT-PCR assays to examine the cardiac abnormalities in mice treated with sodium valproate (NaVP) and determined the effects of NaVP on histone deacetylase activity and the expression of heart development-related genes in mouse myocardial cells.

**Results:**

The experimental data show that NaVP can induce cardiac abnormalities in fetal mice in a dose-dependent manner. NaVP causes a dose-dependent inhibition of hitone deacetylase (HDAC) activity in mouse myocardial cells. However, the expression levels of HDAC (both HDAC1 and HDAC2) are not significantly changed in fetal mouse hearts after administration of NaVP in pregnant mice. The transcriptional levels of other heart development-related genes, such as CHF1, Tbx5 and MEF2, are significantly increased in fetal mouse hearts treated with NaVP.

**Conclusions:**

The study indicates that administration of NaVP in pregnant mice can result in various cardiac abnormalities in fetal hearts, which is associated with an inhibition of histone deacetylase without altering the transcription of this enzyme.

## Introduction

Valproic acid (VPA) has been widely used as an anticonvulsant drug for over 40 years. It is unusual among anticonvulsants in that it has broad activity against both generalized and partial seizures [1]. VPA is relatively free of side-effects compared to other anticonvulsants and is routinely used in epileptic patients [2]. However, studies have indicated a potent teratogenicity of valproic acid, or sodium valproate. VPA has been associated with a variety of major and minor congenital malformations, including a 20-fold increase in neural tube defects, cleft lip and palate, cardiovascular abnormalities, genitourinary defects, and autism. Furthermore, there is an established relationship between VPA dose and adverse outcome [3]. It has been suggested that poly-therapy treatment in epileptic pregnant women increases the risk of teratogenicity in offspring. Maternal VPA use during pregnancy is associated with adverse fetal outcome including cardiac defects and skeletal malformations [4]. The pattern of major malformations, minor dysmorphic features, and neurological abnormalities seen in children prenatally exposed to VPA is referred to as the fetal valproate syndrome [4]. A case study reported a complex cardiac defect with hypoplastic right ventricle in a fetus with valproate exposure [5]. Many animal studies also confirm the teratogenicity of VPA in the animals exposed to the drug [6-8].

Despite its long-standing usage, the mechanism of the anticonvulsant activity of valproate is still controversial. The mechanism underlying VPA-induced side effects and teratogenicity is also unknown. Recently, VPA has been defined as a novel class of histone deacetylase (HDAC) inhibitors, modifying chromatin structure and neuronal gene expression [9-11]. In the present study, we have applied sodium valproate (NaVP) to pregnant mice and investigated cardiac malformation during development. Our results indicate that administration of NaVP in pregnant mice can result in various cardiac abnormalities in fetal hearts, which is likely associated with an inhibition of histone deacetylase without altering the transcription of this enzyme.

## Methods

### Animals

The C57/B6 mice used in this study were maintained as a pathogen-free colony at Florida Atlantic University at Boca Raton, FL. Wild-type (WT) littermates were used as controls in the present study. This investigation was in accordance with the protocols approved by the Institutional Animal Care and Use Committees at Florida Atlantic University. To obtain pregnant mice, female mice were mated with male breeders and inspected every morning for 4 days. Females showing avaginal plug were immediately separated from the males and the morning was denoted as day 1. The pregnant mice were intraperitoneally injected with various amounts of sodium valproate (0, 200, 400, 600 and 700 mg/kg body weight) (Sigma, USA) on day 7 and control group were injected with same volume saline.

### Histology

Fetal hearts isolated from the newborns of sodium valproate (NaVP) treated mice and the saline treated control mice were washed in cold PBS solution. The hearts were immersed in 10% formalin solution for at least 2 h. The hearts were dehydrated progressively in 50% ethanol for 1 h, 70% ethanol for 1 h, in 95% ethanol 1 h and then in 100% ethanol overnight. After xylene treatment, the hearts were embedded in 100% paraffin. Fixed hearts were sectioned into 5-μm thick slices and stained with hematoxylin and eosin. The slides were viewed under an Olympus SZX12 inverted microscope and the images were captured by an Olympus U-CMAD3 camera.

### Mouse myocardium culture

Mouse myocardium separation and culture were carried out using a Neomyt Isolation System for Neonatal Rat/Mouse Cardiomyocytes (Cellutron, MD, USA) according to the protocol from the manufacturer. Briefly, the ventricles from 5 to 7 neonatal mouse hearts (1-3 days after birth) were collected and washed with cold B1. The ventricular tissues were diced and digested for 12 min at 37°C in dissociation buffer B2 + EC. After digestion, the cell pellet was resuspended in B3 plus 50% NS media. The dissociations were repeated 6 times until all tissues were dispersed into single cells. The cells were collected by centrifuging at 1,000 g for 1 min and resuspended in NS media containing 10% of bovine calf serum. Preplating was performed to eliminate the nonmyocytes by incubating the culture dishes at 37°C for 30 min. After 30 min, the cell suspension was plated into 24-well cell culture plates. The cells were cultured for 24 h prior to drug treatment. The characterization of neonatal cardiac myocytes was performed as described in our previous studies [12]. The cardiac myocyte purity of a 48-hour culture was about 80-85% as measured by immunoflurescent staining with monoclonal antibodies against mouse myosin heavy chain. Most of the cultured neonatal cardiac myocytes contracted regularly under microscopic observation and some cells formed synchronously and spontaneously contracting myocardial tissue.

### HDAC activity assay

The cultured mouse neonatal cardiac myocytes (described above) were treated with 0 μM, 10 μM and 1 mM of NaVP for 8 hours before assays. The HDAC activity in NaVP treated and control culture was determined using a HDAC Fluorometric Cellular Activity Assay Kit (Biomol, PA, USA) according to the manufacturer's instructions. Briefly, the culture media in treated and control cardiac myocytes were replaced with 200 μl/well of fresh media containing 200 μM Fluor de Lys Substrate (KI-104). Plates were incubated at 37°C for 1 hour, with each condition represented in triplicate. To terminate HDAC activity and begin development of the fluorescence signal, 200 μl per well of the 1× Developer was added and mixed by up and down pipetting. The mixtures were incubated for an additional 15 min at 37°C, and fluorescence was measured using a SpectraMax M5^e ^Microplate Reader (Molecular Devices, CA, USA), with an excitation wavelength of 360 nm and an emission wavelength of 460 nm.

### Quantitative polymerase chain reaction

The treated and control pregnant mice were sacrificed on day 16 and fetal hearts were isolated and pulverized in liquid nitrogen. Total RNA was extracted using a Tri-Reagent (Sigma, MO, USA) according to the manufacturer's instructions. Residual genomic DNA was removed by treatment with 2 units of rDNase I (Ambion, TX, USA) at 37°C for 1 hour. The DNA-free RNA samples were re-extracted with an equal volume of Tri-Reagent. The aqueous phase containing RNA was precipitated with isopropyl alcohol, and the RNA was dissolved in RNase-free water. 2 μg of extracted total RNA was primed by random primers and reverse transcribed using a ThermoScript RT-PCR System (Invitrogen, CA, USA) at 55°C for 50 min, and then terminated by incubating at 85°C for 5 min. cDNA was kept at -20°C prior to PCR amplification. The specific primers were designed using the Primer Express computer program (Perkin Elmer Applied Biosystems, CA, USA) and all primer sequences were designed to span at least one intron to diminish residue genomic DNA interference. The primer sequences, product lengths and amplification conditions are summarized as follows:

HDAC1, FW: GGGCACCAAGAGGAAAGT; RV: CTCCCGTGGACAACTGA

HDAC2, FW: GACATATGAGACTGCAGTTGC; RV: ACCTCCTTCACCTTCATCCTC

Nkx2.5, FW: CACCCACGCCTTTCTCAGTC; RV: CCATCCGTCTCGGCTTTGT

GATA4, FW: CTGTCATCTCACTATGGGCA; RV: CCAAGTCCGAGCAGGAATTT

Tbx5, FW: CAAACTCACCAACAACCACC; RV: GCCAGAGACACCATTCTCAC

MEF2C, FW: TAATGGATGAGCGTAACAGACAGG; RV: ATCAGACCGCCTGTGTTACC

CHF1, FW: GACAACTACCTCTCAGATTATGGC;

RV: TAGCCACTTCTGTCAAGCACTC

β-actin, FW: CCACTGCCGCATCCTCTTCCTC;

RV: CAGCAATGCCTGGGTACATGGTG

For semi-quantification, PCR reactions were carried in 1 × PCR buffer (Invitrogen, CA, USA), 1.5 mM MgCl_2_, 200 μM dNTP, 0.5 μM of each primer, 0.2 U of Platinum Taq DNA polymerase (Invitrogen) and 2 μl of cDNA template in a total volume of 50 μl. A MasterCycler gradient PCR system (Eppendorf, Hamburg, Germany) was programmed as 1 cycle at 95°C for 2 min, 1 min at 94°C followed by 50 sec at optimized annealing temperature and then followed by 1 min at 72°C for 35 - 45 cycles, and 10 min at 72°C for 1 cycle. β-actin was used as the reference gene. 10 μl of the PCR reaction was resolved in 1.2% agarose gel in TBE buffer, stained with Ethidium Bromide for 1 h and then photographed or scanned. Quantification of amplified products was performed by QuantityOne software (Bio-Rad, PA, USA). The intensities of the interests mRNA bands were normalized relative to that of β-actin bands by dividing the former by the β-actin product densities.

Real-time PCR were performed in 96-well optical PCR plates using a Stratagene Mx3005P Real-Time PCR Systems (Stratagene, CA, USA) to confirm the expressions according to the manufacturer's instructions. Reactions were conducted in a 20 μl volume of reaction mix with 2 μl cDNA, 0.5 μM primers, the optimized MgCl_2 _(1.5-3.0 mM) and 1× Fast Master SYBR Green I (Roche, IN, USA). Assays were duplicated and the specificity of PCR products was checked with a high-resolution gel electrophoresis showing a single product band with the desired sequence length. The analyses of relative mRNA expression were carried out using the 2 ^-ΔΔCt ^method [13].

### Statistical analyses

Statistical significance was determined by ANOVA followed by post hoc Tukey's multiple comparison test. Statistical significance was set at *P *< 0.05.

## Results

**Figure 1:**
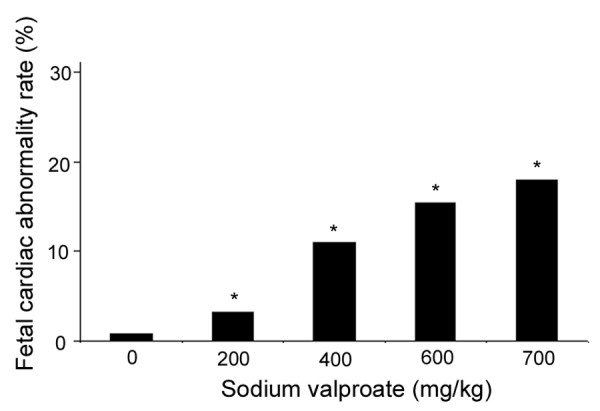
**Dose-dependent cardiac abnormalities in fetal mice prenatally exposed to NaVP**. Cardiac abnormality rate increased significantly in fetal mice (gestation day 19) after prenatal exposure to NaVP (pregnant female mice received a one-time injection of NaVP on gestation day 7 at various doses). The cardiac abnormality caused by NaVP shows a dose-dependent pattern with the highest rate in the group of a treatment dose at 700 mg/kg body weight. * P < 0.05 vs. control without sodium valproate treatment.

**Figure 2:**
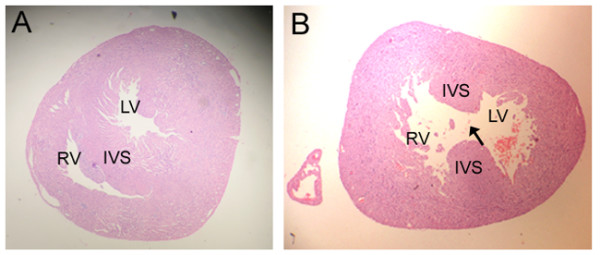
**Histological examination of cardiac sections from fetal hearts with or without exposure to NaVP**. Cardiac sections were examined from the fetal mice (gestation day 19) with or without prenatal exposure to NaVP (exposure group, the pregnant female mice received a one-time injection intraperitoneally of NaVP on gestation day 7 at a dose of 700 mg/kg body weight; control group, the pregnant female mice received a one-time injection of saline solution at the same time). (A) A representative cross section image of normal fetal hearts from the control groups. (B) A representative image of the fetal hearts from the NaVP treated group showing an interventricular septal interruption indicated by an arrow. RV, right ventricle; LV, left ventricle; IVS, interventricular septum.

The results from teratogenesis analysis indicated that the average fetus number per pregnant female mouse was not significantly different between the treatment group with NaVP and the control groups, however, the fetal death and fetal resorption rates were significantly higher in all NaVP treated groups (D6 to D9) compared to the control groups (Table 1). In addition, NaVP treatment caused a significant increase of cardiac abnormality rate in the treatment groups compared to the controls, with the highest cardiac abnormality rate in the group treated with NaPV on gestation day 7 (D7)(Table 1). Cardiac abnormalities in fetal mice exposed to NaVP showed a dose-dependent pattern with the highest rate in the group treated with 700 mg/kg NaVP in our assay conditions (Figure 1). Histological examination of cardiac sections from both treated and control groups indicated that the cardiac abnormalities were characterized primarily by interventricular septal defects and by atrial septal defects as well as other types of congenital heart diseases. Figure 2 shows representative cardiac sections after HE staining from the control group (Figure 2A) and the NaVP treated group (Figure 2B). Figure 2B clearly shows a tissue loss in interventricular septum (IVS), as indicated by an arrow, between the right ventricle (RV) and the left ventricle (LV) of the heart, whereas the IVS in the heart of the control group is intact without any damage (Figure 2A).

**Table 1:** Fetal loss and cardiac abnormality in NaVP exposed dams

Treatment (Gestation)	No. of dams	Total No. of Live fetuses (fetus/dam)	N. of death or resorption	Cardiac abnormalities	
				
				No. in live fetus	%
Day 6					
Control	10	163 (16.3)	2	1	0.60
Treated	20	148 (7.4)*	63*	13	8.70*
					
Day 7					
Control	12	148 (12.3)	3	1	0.67
Treated	24	162 (6.75)*	90*	34	20.90*
					
Day 8					
Control	10	147 (14.7)	3	0	0.00
Treated	20	132 (6.6)*	78*	13	9.80*
					
Day 9					
Control	10	149 (14.9)	1	1	0.68
Treated	20	142 (7.1)*	60*	9	6.30*

Pregnant female mice received a one-time injection (i.p.) of NaVP (700 mg/kg body weight) on gestation day as indicated. The fetuses were examined on gestation day 19 after sacrifice of the pregnant mice as described in Materials and Methods.

* *P *< 0.05 compared to controls.

**Figure 3:**
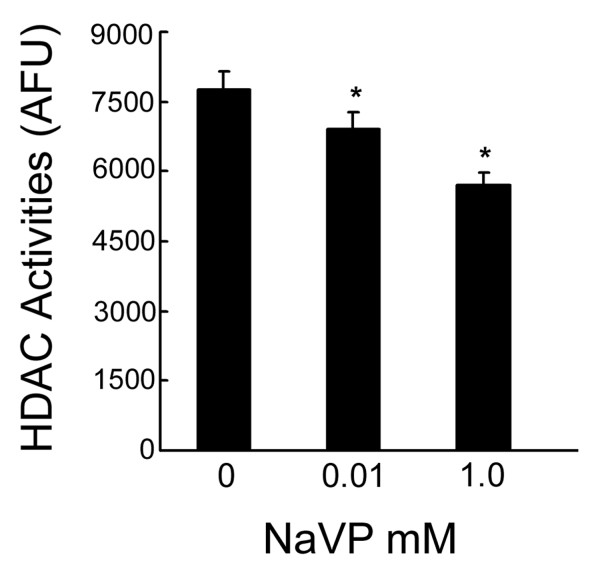
**Inhibition of NACD activities by NaVP in cardiac myocytes**. HDAC activities were measured as described in Materials and Methods and HDAC activities were presented as absorbance fluorescent unit (AFU). Values are Mean ± SEM from 3 separated experiments. * P < 0.05 vs. control without sodium valproate treatment.

Since acetylation of histones H3 and H4 in mammalian cell nucleus plays a critical role in gene expression and organ development, we further examined the effect of NaVP on histone deacetylase (HDAC) in cultured neonatal mouse cardiac myocytes. Figure 3 shows that NaVP inhibits HDAC activity in mouse cardiac myocytes in a dose-dependent manner. Even at a low dose of 10 μM, NaVP caused a significant inhibition of HDAC activity in these cells (P < 0.05)(Figure 3).

**Figure 4:**
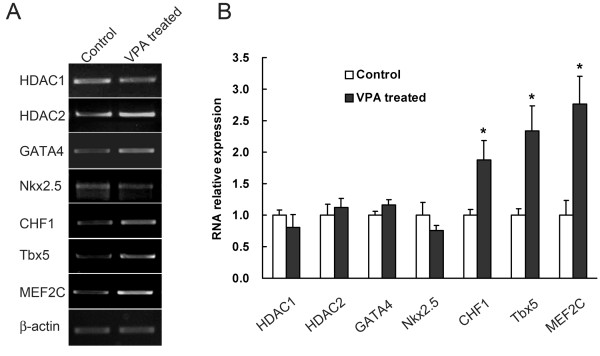
**Effect of NaVP on transcriptional regulation of various cardiac development-related genes**. Gene expression levels were analyzed using RT-PCR techniques as described in Materials and Methods. (A) A representative RT-PCR analysis of HDAC1, HDAC2, MEF2C, CHF1, GATA4, Nkx2.5 CHF1, Tbx5 and MEF2C mRNA expression in E16 fetal hearts with NaVP treatment on E7. Saline treated mice hearts served as controls. β-actin was used as an internal control. (B) Summary of real-time PCR data from 3 separate experiments. The values from each sample were normalized to that of β-actin mRNA level. Values are presented as means ± SEM of triplicate experiments. * P < 0.05 vs. controls.

We carried out quantitative RT-PCR experiments on fetal hearts to determine the mechanism of NaVP-induced HDAC inhibition. The mRNA expression of various genes in the heart was determined including HDAC1, HDAC2 and other heart development related genes, such as GATA4, CHF1, Tbx5, MEF2C, etc. Figure 4 shows that no significant changes are observed in the expression levels of HDAC1, HDAC2, GATA4, Nkx2.5 between NaVP-treated groups and the control groups. The data indicate that the inhibitory effect of NaVP on HDAC activities is unlikely to act through the transcriptional inhibition of this gene. In addition, our data showed that the expression levels were significantly increased in other tested genes, such as CHF1, Tbx5 and MEF2C in fetal hearts from NaVP-treated groups compared to that of the controls (Figure 4).

## Discussion

Valproic acid was considered a relatively side-effect free compound and was routinely used in epileptic patients, in come cases successfully for decades [2]. However, its side-effect, especially its teratogenecity is attracting more and more attention. The name of the fetal valproate syndrome indicates that multiple organ malformations have been seen in children prenatally exposed to valproic acid [4]. In our study, we further confirm the teratogenetic effects of NaVP on fetuses when pregnant mice are exposed to this compound during the gestation days of organ formation, i.e. gestation day 6 to day 9 in mice. A hypoplastic right ventricle has been reported in a fetus with valproate exposure [5]. Our study indicates that prenatal NaVP exposure causes significant cardiac abnormalities, in particular, interventricular septal defects in the heart.

Histones form the protein core of nucleosomes, the DNA/protein complexes, which are the subunits of eukaryotic chromatin. The chromatin histone acetylation/deacetylation plays a critical role in the protein gene expression and organogenesis [14]. The balance of histone acetylation/deacetylation is controlled by two key groups of enzymes: histone acetyltransferases (HATs) catalyze the acetylation of specific histone lysine residues and histone deacetylases (HDACs) are responsible for hydrolytic removal of these acetyl groups [15-17]. Histone hyperacetylation correlates with an open, decondensed chromatin structure and gene activation, while hypoacetylation correlates with chromatin condensation and transcriptional repression. Consistent with this, HATs have been shown to associate with several transcription activators and some transcription activators have been found to have intrinsic HAT activity [15-17]. Conversely, HDACs are found to associate with transcription repression complexes [18].

To further explore the mechanism underlying valproate induced cardiac malformation, we performed experiments to determine the effect of valproate on histone deacetylase activity. Our data indicate that valproate can significantly inhibit HDAC activity in mouse cardiac myocytes exposed to NaVP. This result is consistent with a recent report that valprotic acid is defined as a novel class of HDAC inhibitors inducing differentiation of transformed cells [9]. The mechanism underlying the inhibitory action of valproate on HDAC is still unknown. According to our experimental data, we believe that valproate does not inhibit HDAC activities through the intervention of the transcription of HDAC genes. More likely, valproate inhibits HDAC activities directly by binding with the active site of the enzyme as other HDAC inhibitors do [19-21].

Although no significant changes were observed in the expression levels of HDACs, GATA4 and Nkx2.5 in fetal hearts exposed to NaVP, some significant changes were observed in the expression levels of several heart development-related transcription factors such as CHF1, MEF2C and Tbx5 in fetal hearts exposed to NaVP. CHF1 is a member of the cardiovascular basic helix-loop-helix factor family and plays an important role in regulation of ventricular septation in mammalian heart development [22,23]. MEF2C is a member of the MEF2 family of transcription factors that bind a conserved A-T-rich DNA sequence associated with most cardiac muscle structural genes and are expressed in cardiogenic precursor cells and differentiated cardiac myocytes during embryogenesis [24,25]. Tbx5 is a T-box transcription factor that plays a critical role in cardiac development. Tbx5 is expressed in the developing heart in vertebrate embryos during critical stages of morphogenesis and patterning. In human, mutations in the Tbx5 gene have been associated with Holt-Oram syndrome, which is characterized by developmental anomalies in the heart and forelimbs [26,27]. The expression pattern of Tbx5 in the heart is very interesting. It is uniformly expressed throughout the entire cardiac crescent early in the developing heart. With the development of the heart, Tbx5 is asymmetrically expressed in the heart. Expression of Tbx5 in the ventricular septum is restricted to the left side and is contiguous with left ventricular free wall expression. Some studies indicate that these patterns of Tbx5 expression provide an embryologic basis for the prevalence of atrial and ventricular septal defects observed in patients with Holt-Oram syndrome [28]. Liberatore *et al *generated transgenic mouse embryos that over-express Tbx5 throughout the primitive heart tube and found a significant loss of ventricular-specific gene expression and retardation of ventricular chamber morphogenesis in these embryos, indicating that Tbx5 plays an essential role in early heart morphogenesis and chamber-specific gene expression [29]. However, it remains unclear for the moment as to why the expression of these genes is enhanced by valproate and what the relationship is between valproate-mediated increase of these gene expression and valproate-induced cardiac malformation during heart development. Further studies are on the way to explore the down-stream activities after the valproate-enhanced expression of these transcriptional factors in the developing heart.

## Conclusion

Our data indicate that administration of NaVP in pregnant mice can result in various cardiac abnormalities in fetal hearts, which is likely associated with an inhibition of histone deacetylase without altering the transcription of this enzyme.

## Competing interests

The authors declare that they have no competing interests.

## Authors' contributions

GW carried out the teratogenecity experiments in mice and performed histological examinations. CN designed the primers and carried out the molecular genetic studies using RT-PCR techniques. JCR edited and revised the manuscript. XH organized the design of the study and manuscript preparation. JT participated in study design and coordination.

All authors read and approved the final manuscript.

## References

[B1] Peterson GM Naunton M Valproate: a simple chemical with so much to offer J Clin Pharm Ther 2005 30 417 421 10.1111/j.1365-2710.2005.00671.x 16164485

[B2] Gerstner T Bell N Konig S Oral valproic acid for epilepsy-long-term experience in therapy and side effects Expert Opin Pharmacother 2008 9 285 292 10.1517/14656566.9.2.285 18201150

[B3] Alsdorf R Wyszynski DF Teratogenicity of sodium valproate Expert Opin Drug Saf 2005 4 345 353 10.1517/14740338.4.2.345 15794725

[B4] Kozma C Valproic acid embryopathy: report of two siblings with further expansion of the phenotypic abnormalities and a review of the literature Am J Med Genet 2001 98 168 175 10.1002/1096-8628(20010115)98:2<168::AID-AJMG1026>3.0.CO;2-O 11223853

[B5] Ten Berg K van Oppen ACC Nikkels PGJ Groot ACG Voet GB van der Brilstra EH Lindhout D Complex cardiac defect with hypoplastic right ventricle in a fetus with valproate exposure Prenatal Diagn 2005 25 156 158 10.1002/pd.1098 15712340

[B6] Sonoda T Ohdo S Ohba K Okishima T Hayakawa K Sodium valproate-induced cardiovascular abnormalities in the jcl:ICR mouse fetus: peak sensitivity of gestational day and dose-dependent effect Teratology 1993 48 127 132 10.1002/tera.1420480206 8211818

[B7] Menegola E Broccia ML Nau H Prati M Ricolfi R Giavini E Teratogenic effects of sodium valproate in mice and rats at midgestation and at term Teratog Carcinog Mutagen 1996 16 97 108 10.1002/(SICI)1520-6866(1996)16:2<97::AID-TCM4>3.0.CO;2-A 8875740

[B8] Dura S Ceylan S Ceylan S Comparative effects of valproic acid sodium for Chiari-like malformation at 9 and 10 days of gestation in the rat Childs Nerv Syst 2001 17 399 404 10.1007/s003810000417 11465793

[B9] Daniels T Gallagher M Tremblay G Rodgers RL Effects of valproic acid on cardiac metabolism Can J Physiol Pharmacol 2004 82 927 933 10.1139/y04-096 15573154

[B10] Gottlicher M Minucci S Zhu P Kramer OH Schimpf A Giavara S Sleeman JP Lo Coco F Nervi C Pelicci PG Heinzel T Valproic acid defines a novel class of HDAC inhibitors inducing differentiation of transformed cells EMBO J 2001 20 6969 6978 10.1093/emboj/20.24.6969 11742974 PMC125788

[B11] Nalivaeva NN Belyaev ND Turner AJ Sodium valproate: an old drug with new roles Trends in Pharmacol Sci 2009 30 509 513 10.1016/j.tips.2009.07.002 19762089

[B12] Riedel B Jia Y Du J Akerman S Huang XP Thyroid hormone inhibits slow skeletal TnI expression in cardiac TnI-null myocardial cells Tissue & Cell 2005 37 47 51 10.1016/j.tice.2004.10.002 15695175

[B13] Livak KJ Schmittgen TD Analysis of relative gene expression data using real-time quantitative PCR and the 2(-Delta Delta C(T)) method Methods 2001 25 402 408 10.1006/meth.2001.1262 11846609

[B14] Eberharter A Becker PB Histone acetylation: a switch between repressive and permissive chromatin EMBO Rep 2002 3 224 229 10.1093/embo-reports/kvf053 11882541 PMC1084017

[B15] Grunstein M Histone acetylation in chromatin structure and transcrition Nature 1997 389 349 352 10.1038/38664 9311776

[B16] Ng HH Bird A Histone deacetylases: silencers for hire Trends Biochem Sci 2000 25 121 126 10.1016/S0968-0004(00)01551-6 10694882

[B17] Cheung WL Briggs SD Allis CD Acetylation and chromosomal functions Curr Opin Cell Biol 2000 12 326 333 10.1016/S0955-0674(00)00096-X 10801466

[B18] Rundlett SE Carmen AA Suka N Turner BM Grunstein M Transcriptional repression by UME6 involves deacetylation of lysine 5 of histone H4 by RPD3 Nature 1998 392 831 835 10.1038/33952 9572144

[B19] Finnin MS Donigian JR Cohen A Richon VM Rifkind RA Marks PA Breslow R Pavletich NP Structures of a histone deacetylase homologue bound to the TSA and SAHA inhibitors Nature 1999 401 188 195 10.1038/43710 10490031

[B20] Somoza JR Skene RJ Katz BA Mol C Ho JD Jennings BA Luong C Arvai A Buggy JJ Chi E Tang J Sang B Verner E Wynands R Leahy EM Dougan DR Snell G Navre M Knuth MW Swanson RV McRee DE Tari LW Structural snapshots of human HDAC8 provide insights into the class I histone deacetylases Structure 2004 12 1325 1334 10.1016/j.str.2004.04.012 15242608

[B21] Vannini A Volpari C Filocamo G Casavola EC Brunetti M Renzoni D Chakravarty P Paolini C Francesco R Gallinari P Steinkuhler C Di Marco S Crystal structure of a eukaryotic zinc-dependent histone deacetylase, human HDAC8, complexed with a hydroxamic acid inhibitor Proc Natl Acad Sci USA 2004 101 15064 15069 10.1073/pnas.0404603101 15477595 PMC524051

[B22] Sakata Y Kamei CN Nakagami H Bronson R Liao JK Chin MT Ventricular septal defect and cardiomyopathy in mice lacking the transcription factor CHF1/Hey2 Proc Natl Acad Sci USA 2002 99 16197 16202 10.1073/pnas.252648999 12454287 PMC138588

[B23] Sakata Y Koibuchi N Xiang F Youngblood JM Kamei CN Chin MT The spectrum of cardiovascular anomalies in CHF1/Hey2 deficient mice reveals roles in endocardial cushion, myocardial and vascular maturation J Mol Cell Cardiol 2006 40 267 273 10.1016/j.yjmcc.2005.09.006 16242143

[B24] Lin Q Schwarz J Bucana C Olson EN Control of mouse cardiac morphogenesis and myogenesis by transcription factor MEF2C Science 1997 276 1404 1407 10.1126/science.276.5317.1404 9162005 PMC4437729

[B25] van Oort RJ van Rooij E Bourajjaj M Schimmel J Jansen MA Nagel R van der Doevendans PA Schneider MD van Echteld CJA De Windt LJ MEF2 activates a genetic program promoting chamber dilation and contractile dysfunction in calcineurin-induced heart failure Circulation 2006 114 298 308 10.1161/CIRCULATIONAHA.105.608968 16847152

[B26] Basson CT Bachinsky DR Lin RC Levi T Elkins JA Soults J Grayzel D Kroumpouzou E Traill TA Leblanc-Straceski J Renault B Kucherlapati R Seidman JG Seidman CE Mutations in human TBX5 cause limb and cardiac malformation in Holt-Oram syndrome Nat Genet 1997 15 30 35 10.1038/ng0197-30 8988165

[B27] Li QY Newbury-Ecob RA Terrett JA Wilson DI Curtis AR Yi GH Gebuhr T Bullen PJ Robson SC Strachan T Bonnet D Lyonnet S Young ID Raeburn JA Buckler AJ Law DJ Brook JD Holt-Oram syndrome is caused by mutations in TBX5, a member of the Brachyury (T) gene family Nat Genet 1997 15 21 29 10.1038/ng0197-21 8988164

[B28] Bruneau BG Logan M Davis N Levi T Tabin CJ Seidman JG Seidman CE Chamber-specific cardiac expression of Tbx5 and heart defects in Holt-Oram syndrome Developmental Biol 1999 211 100 108 10.1006/dbio.1999.9298 10373308

[B29] Liberatore CM Searcy-Schrick RD Yutzey KE Ventricular expression of tbx5 inhibits normal heart chamber development Developmental Biol 2000 223 169 180 10.1006/dbio.2000.9748 10864469

